# On the Discovery, Biological Effects, and Use of Cisplatin and Metallocenes in Anticancer Chemotherapy

**DOI:** 10.1155/2012/140284

**Published:** 2012-07-12

**Authors:** Santiago Gómez-Ruiz, Danijela Maksimović-Ivanić, Sanja Mijatović, Goran N. Kaluđerović

**Affiliations:** ^1^Departamento de Química Inorgánica y Analítica, E.S.C.E.T., Universidad Rey Juan Carlos, 28933 Móstoles, Spain; ^2^Institute for Biological Research “Sinisa Stankovic”, University of Belgrade, Boulevard of Despot Stefan 142, 11060 Belgrade, Serbia; ^3^Institut für Chemie, Martin-Luther-Universität Halle-Wittenberg, Kurt-Mothes-Straße 2, 06120 Halle, Germany

## Abstract

The purpose of this paper is to summarize mode of action of cisplatin on the tumor cells, a brief outlook on the metallocene compounds as antitumor drugs as well as the future tendencies for the use of the latter in anticancer chemotherapy. Molecular mechanisms of cisplatin interaction with DNA, DNA repair mechanisms, and cellular proteins are discussed. Molecular background of the sensitivity and resistance to cisplatin, as well as its influence on the efficacy of the antitumor immune response was evaluated. Furthermore, herein are summarized some metallocenes (titanocene, vanadocene, molybdocene, ferrocene, and zirconocene) with high antitumor activity.

## 1. Cisplatin

**Figure 1:**
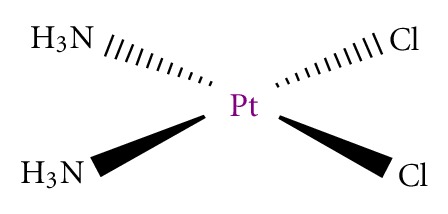
Cisplatin.

Since 1845, when Italian doctor Peyrone synthesized cisplatin (Figure 1), through Rosenberg's discovery of cisplatin antiproliferative potential [1], and subsequent approval for clinical usage in 1978, this drug is considered as most promising anticancer therapeutic [2, 3]. Cisplatin is highly effective against testicular, ovarian, head and neck, bladder, cervical, oesophageal as well as small cell lung cancer [4]. 

**Figure 2:**
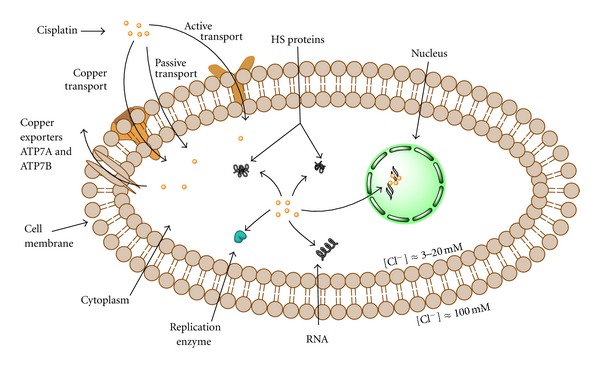
Cisplatin and the cell: transport/export and targets.

For more than 150 years, first exaltation about this “drug of the 20th century” was replaced with discouraging data about its toxicity and ineffectiveness got from clinical practice. It was found that cisplatin induced serious side effects such as nephrotoxicity, neurotoxicity, ototoxicity, nausea, and vomiting [5]. General toxicity and low biological availability restricted its therapeutically application. In addition, it is known that some tumors such as colorectal and nonsmall lung cancers are initially resistant to cisplatin while other like ovarian and small cell lung cancers easily acquired resistance to drug [6]. Numerous examples from *in vitro* studies confirmed that exposure to cisplatin often resulted in development of apoptotic resistant phenotype [7–9]. Following this, development of cisplatin resistant cell lines is found useful for testing the efficacy of future cisplatin modified drugs and on the other hand for evaluation of mechanisms involved in development of resistance. For better understanding of unresponsiveness to cisplatin, it is necessary to define the exact molecular targets of drug action from the moment of entering tumor cell. It is proposed the intact cisplatin which avoided bounding to plasma proteins enter the cell by diffusion or active transport via specific receptors (Figure 2) [10, 11]. Cisplatin is able to use copper-transporting proteins to reach intracellular compartments [12–14]. In addition, regarding to its chemical reactivity, cisplatin can influence cell physiology even through interaction with cell membrane molecules such as different receptors.

### 1.1. Cisplatin and DNA

**Figure 3:**
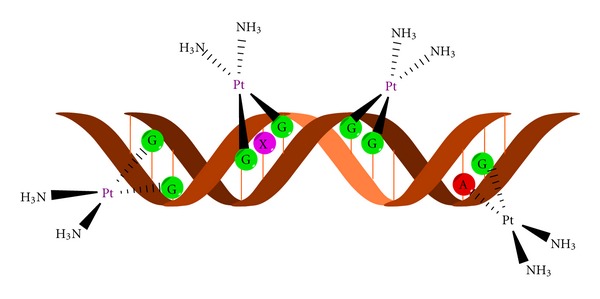
DNA adduct formation with cisplatin moiety.

Although it is known that DNA is a major target for cisplatin, only 5–10% intracellular concentration of cisplatin is found in DNA fraction while 75–85% binds to nucleophilic sites of intracellular constituents like thiol containing peptides, proteins, replication enzymes, and RNA [6, 15–17]. This preferential binding to non-DNA targets offers the explanation for cisplatin resistance but also its high toxicity. Prerequisite of efficient formation of cisplatin DNA adducts is hydratization of cisplatin enabled by low chloride ions content inside the cells [18]. N7 of guanine and in less extend adenine nucleotide are targeted by platinum [19]. Binding of cisplatin to DNA is irreversible and structurally different adducts are formed. The adducts are classified as intrastrand crosslinking of two nucleobases of single DNA strand, interstrand crosslinking of two different strands of one DNA molecule, chelate formation through *N*- and *O*-atoms of one guanine, and DNA-protein crosslinks [20, 21]. Cisplatin forms about 65% pGpG-intrastrand crosslinks, 25% pApG-intrastrand crosslinks, 13% interstrand or intrastrand crosslinks on pGpXpG sequences, and less than 1% of monofunctional adducts (Figure 3) [22]. Crucial role of 1,2-intrastrand crosslinks in antitumor potential of the cisplatin is supported by two facts. First, high mobility group proteins (HMG) specifically recognize this type of cisplatin-DNA interaction and second, these adducts are less efficiently removed by repair enzymes [17]. In addition, important mediators of cisplatin toxicity are ternary DNA-platinum-protein crosslinks (DPCL) whose frequency is dependent from the cell type as well as the type of the treatment. DPCLs inhibited DNA polymerization or their own removal by nucleotide excision repair system more potently than other DNA adducts [17]. In fact, cisplatin DNA adducts can be repaired by nucleotide excision repair proteins (NER), mismatch repair (MMR), and DNA-dependent protein kinases protein [17].

### 1.2. DNA Repair Mechanism

Nucleotide excision repair proteins are ATP-dependent multiprotein complex able to efficiently repair both inter as well as intrastrand DNA-cisplatin adducts. Successful repair of 1,2-d(GpG) and 1,3-d(GpNpG) intrastrand crosslinks has been found in different human and rodent NER systems [23, 24]. This repair mechanism is able to correct the lesions promoted by chemotherapeutic drugs, UV radiation as well as oxidative stress [17]. Efficacy of NER proteins varying in different type of tumors and is responsible for acquirement of cisplatin resistance. Low level of mentioned proteins is found in testis tumor defining their high sensitivity to cisplatin treatment. Oppositely, ovarian, bladder, prostate, gastric, and cervical cancers are resistant to cisplatin based therapy due to overexpression of several NER genes [25, 26].

Mismatch repair (MMR) proteins are the post replication repair system for correction of mispaired and unpaired bases in DNA caused by DNA Pt adducts. MMR recognized the DNA adducts formed by ligation of cisplatin but not oxaliplatin [27–30]. Defective MMR is behind the resistance of ovarian cancer to cisplatin and responsible for the mutagenicity of cisplatin [31]. 

DNA dependent protein kinase is a part of eukaryotic DNA double strand repair pathway. This protein is involved in maintaining of genomic stability as well as in repair of double strand breaks induced by radiation [31]. In ovarian cancer presence of cisplatin DNA adducts inhibited translocation of DNA-PK subunit Ku resulting in inhibition of this repair protein [32].

Special attention is focused on recognition of cisplatin-modified DNA by HMG proteins (HMG). It is hypothesized that HMG proteins protected adducts from recognition and reparation [17, 31]. Moreover, it was postulated that these proteins modulate cell cycle events and triggered cell death as a consequence of DNA damage. One of the members from this group marked as HMGB1 is involved in MMR, increased the p53 DNA-binding activity and further stimulated binding of different sequence specific transcription factors [33]. Few studies revealed that cisplatin sensitivity was in correlation with HMGB level, while other studies eliminated its significance in response to cisplatin treatment. Contradictory data about the relevance of HMG proteins in efficacy of cisplatin therapy indicated that this relation is defined by cell specificity.

### 1.3. Cytotoxicity of Cisplatin

Other non-HMG nuclear proteins are also involved in cytotoxicity of cisplatin. Presence of cisplatin DNA adducts is able to significantly change or even disable the primary function of nuclear proteins essential for transcription of mammalian genes (TATA binding protein, histon-linker protein H1 or 3-methyladenine DNA glycosylase mammalian repair protein) [34–36].

Although cytotoxicity of cisplatin is usually attributed to its reactivity against DNA and subsequent lesions, the fact that more than 80% of internalized drug did not reach DNA indicated the involvement of numerous non-DNA cellular targets in mediation of cisplatin anticancer action [6]. As a consequence of exposure to cisplatin, different signaling pathways are affected. There is no general concept applicable to all types of tumor. It is evident that response to cisplatin is defined by cell specificity. Numerous data revealed changes in activity of most important signaling pathways involved in cell proliferation, differentiation and cell death such as PI3K/Akt, MAPK as well as signaling pathways involved in realization of death signals dependent or independent of death receptors [33]. It is very important to note that alteration in signal transduction upon the cisplatin treatment could be the consequence of both, DNA damage or interaction with exact protein or protein which is relevant for appropriate molecular response. Some of the interactions between protein and cisplatin are already described. Therefore, it was found that cisplatin directly interacts with telomerase, an enzyme that repairs the ends of eukaryotic chromosomes [31, 37]. In parallel, cisplatin-induced damage of telomeres which are not transcribed and therefore hidden from NER. Other important protein targeted by cisplatin is small, tightly folded molecule known as ubiquitin (Ub) [38]. Ub is implicated in selective degradation of short-lived cellular proteins [39]. It has been hypothesized that direct interaction of cisplatin with this protein presented a strong signal for cell death [40]. Two binding sites were identified as target for cisplatin ligation: *N*-terminal methionine (Met1) and histidine at position 68, while the drug makes at least four types of adducts with protein [38]. This resulted in disturbed proteasomal activity and further cell destruction. Having in mind that proteasomal inactivation by specific inhibitors showed promising results in cancer treatment, this aspect of cisplatin reactivity can be leading cytotoxic effect even to be more powerful than DNA damage [41]. One of the crucial molecules involved in propagation of apoptotic signal through depolarization of mitochondrial potential—cytochrome c is also targeted by cisplatin on Met65 [42]. Further, on the list of protein or peptide targets for cisplatin are glutathione and metallothioneins, superoxide dismutase, lysozyme as well as extracellular protein such as albumin, transferrin, and hemoglobin [43]. Some of mentioned interactions served as drug intracellular pool while their biological relevance is still under investigation. 

### 1.4. Activation of Signaling Pathways Induced with Cisplatin

DNA damage induced by cisplatin represent strong stimulus for activation of different signaling pathways. It was found that AKT, c-Abl, p53, MAPK/JNK/ERK/p38 and related pathways respond to presence of DNA lesions [31, 33]. AKT molecule as most important Ser/Thr protein kinase in cell survival protects cells from damage induced by different stimuli as well as cisplatin [44]. Cisplatin downregulated XIAP protein level and promoted AKT cleavage resulting in apoptosis in chemosensitive but not in resistant ovarian cancer cells [45, 46]. Recently published data about synergistic effect of XIAP, c-FLIP, or NFkB inhibition with cisplatin are mainly mediated by AKT pathway [47].

Protein marked as the most important in signaling of the DNA damage is c-Abl which belongs to SRC family of non-receptor tyrosine kinases [31, 33]. This molecule acts as transmitter of DNA damage triggered by cisplatin from nucleus to cytoplasm [48]. Moreover, sensitivity to cisplatin induced apoptosis is directly related with c-Abl content and could be blocked by c-Abl overexpression [33]. Key role of c-Abl in propagation of cisplatin signals is confirmed in experiments with ABL deficient cells [49]. It was found that cisplatin failed to activate p38 and JNK in the absence of c-Abl. Homology of this kinases with HMGB indicated the possibility that c-Abl recognized and interact with cisplatin DNA lesions like HMGB1 protein [31].

### 1.5. The Role of the Functional p53 Protein

Evaluation of a 60 cell line conducted by the National Cancer Institute revealed that functional p53 protein is very important for successful response to cisplatin treatment [33]. This tumor suppressor is crucial for many cellular processes and determined the balance between cell cycle arrest as a chance for repair and induction of apoptotic cell death [33]. However, despite extensive NCI study, there are controversial data about correlation between cisplatin sensitivity and p53. For example, it was found that functional p53 was associated with amplified cisplatin sensitivity in SaOS-2 osteosarcoma cells in high serum growth conditions while the opposite relation was observed upon starvation [33]. This phenomenon could be connected to autophagic process triggered in serum deficient conditions, which in turn downregulate cisplatin promoted apoptosis [50]. In some other studies, the response to cisplatin was not influenced by p53. It is indicative that antitumor potential of cisplatin and its interaction with p53 is a question of multiple factors such as tumor cell type, specific signaling involved in cancerogenesis, as well as other genetic alterations. In addition, protein involved or influenced by p53 pathway such as Aurora kinase A, cyclin G, BRCA1 as well as proapoptotic or antiapoptotic mediators are also able to control cisplatin toxicity [33]. 

### 1.6. Relation between Cisplatin and Mitogen-Activated Protein (MAP) Kinases

Finally, signaling pathways mediated by mitogen-activated kinases are strongly influenced by cisplatin. These enzymes are highly important in definition of cellular response to applied treatment because they are the major regulators of cell proliferation, differentiation, and cell death. ERK (extracellular signal-related kinase) preferentially responds to growth factor and cytokines but also determines cell reaction to different stress conditions, particularly, oxidative [33]. Cisplatin treatment mainly activated ERK in a dose- and time-dependent manner [33, 51, 52]. However, like as previously described, changes in ERK activity upon the exposure to cisplatin varying from type to type of the malignant cell and is defined by their intrinsic features. Following this, in some circumstances ERK activation antagonized cisplatin toxicity. In cells with significant upregulation of ERK activity in response to cisplatin treatment, exposure to specific MEK1 inhibitor PD98052 abrogated its toxicity. Also, development of the resistance to the cisplatin in HeLa cells is connected with reduced ERK response to the treatment [52]. Moreover, combined treatment with some of the naturally occurring compounds such as aloe-emodin-neutralized cisplatin toxicity through inhibition of ERK, indicated possible negative outcome of combining of conventional and phytotherapy [53, 54]. 

Regardless of numerous evidences about its critical role in cisplatin-mediated cell death, ERK is not the only molecule from MAP family which responded to cisplatin. Several studies revealed JNK (c-Jun N-terminal kinase) activation upon the cisplatin addition [55, 56]. However, similarly to other molecules previously mentioned this signal is not the unidirectional and could be responsible for realization but also protection from death triggered by the cisplatin [57, 58]. Finally, there are numerous evidences about highly important role of third member of MAP kinases, p38, in response to cisplatin [59, 60]. Lack of p38 MAPK leads to appearance of resistant phenotype in human cells [55, 60]. Early and short p38 activation is principally described in cells unresponsive to cisplatin while long-term activation was found in sensitive clones. Moreover, in the light of the fact that this kinase has a role in modifying the chromatin environment of target genes, its involvement in cisplatin-induced phosphorilation of histon 3 was determined [61]. 

### 1.7. On the Mode of Cell Death Induced by Cisplatin

**Figure 4:**
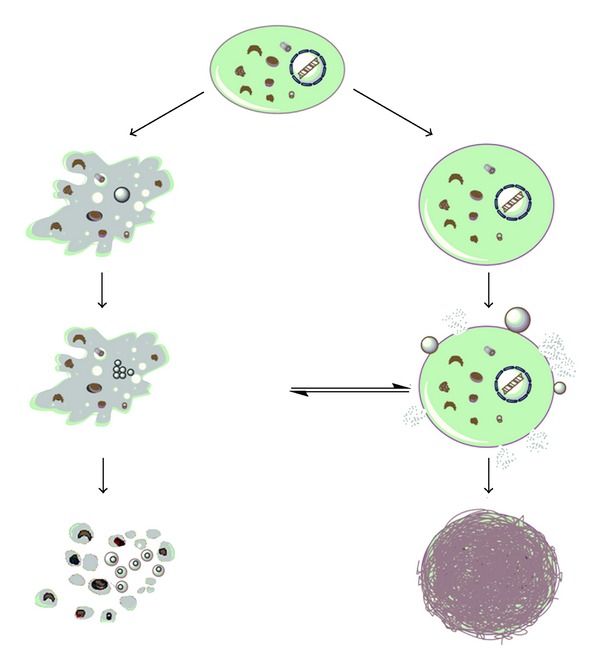
Mode of cell death induced by cisplatin: apoptosis (left) and necrosis (right).

The net effect of intracellular interaction of cisplatin with DNA and non-DNA targets is the cell cycle arrest and subsequent death in sensitive clones. There are two type of death signals resulting from cellular intoxication by this drug (Figure 4). Fundamentally, the drug concentration presents the critical point for cell decision to undergo apoptotic or necrotic cell death [62]. Primary cultures of proximal tubular cells isolated from mouse died by necrosis if they were exposed to high doses of cisplatin just for a few hours while apoptotic cell death is often triggered by long-term exposure to significantly lower concentrations [63]. However, the presence of necrosis in parallel with apoptosis in tumor-cell population indicated that type of cell death is not just the question of dose but also is defined by cell intrinsic characteristics and energetic status of each cell at the moment of the treatment. In fact, it was considered that intracellular ATP level dictate cell decision to die by necrotic or apoptotic cell death [64, 65]. One of the signals which are provoked with DNA damage is PARP-1 activation and subsequent ATP depletion caused by PARP-1 mediated cleavage of NAD+. This event is a trigger for necrotic cell death. However, activated caspases cleaved the PARP-1, preventing necrotic signal and favor the execution of apoptotic process. On the other hand, the inhibition of caspases by intracellular inhibitors IAP together with continual PARP activity and ATP depletion resulted in necrosis [31]. As numerous biological phenomena, this one is not unidirectional. It was found that failure in PARP cleavage may also serve to apoptosis [66]. This paradox was ascribed to changes in pyridine nucleotide pool as well as in pool of ATP/ADP responsible for regulation of mitochondrial potential [67]. Atypical apoptosis was observed in L1210 leukemia cell line exposed to cisplatin. Different death profiles in cisplatin treated cells confirmed plasticity of signals involved in cell destruction and focus the attention to the molecules responsible for resistance to death as possible targets for the therapy. Having in mind that cisplatin is toxic agent against whom the cell can activate autophagy as protective process; the specific inhibition of autophagy by certain type of molecules could amplify the effectiveness of cisplatin [50]. 

### 1.8. Cisplatin in Immune Senzitization

One of the rarely mentioned but very important aspects of antitumor activity of cisplatin is based on the experimental data about its potential to amplified the sensitivity of malignant cells to one or the most potent and selective antitumor immune response mediated by TNF-related apoptosis inducing ligand TRAIL [68, 69]. This molecule is produced by almost all immune cells involved in nonspecific as well as adoptive immune response. Unfortunately, in the moment when tumor is diagnosed, its sensitivity to natural immunity is debatable. In most of the situations, malignant cells became resistant to TRAIL-mediated cytotoxicity [70]. Moreover, it was confirmed that cisplatin promoted their sensitivity to TRAIL. Nature of its immune sensitizing potential is at least partly due to upregulation of expression of TRAIL receptors—DR4 and DR5 on the cellular membrane glioma, colon and prostate cell lines as well as downregulation of cellular form of caspase 8 inhibitor FLIP [68, 69]. In addition, presence of cysteine rich domen in the structure of TRAIL specific death receptors indicated possibility that cisplatin directly interact with them.

### 1.9. Resistance to Cisplatin and How to Surmount It

Resistance to cisplatin could be established at multiple levels, from cellular uptake of the drug through interaction with protein and DNA and finally activation of signals which lead the cell to death. Disturbed drug uptake, drug scavenging by cellular proteins, upregulation of prosurvival signals together with upregulated expression of antiapoptotic molecules such as Bcl-2 and BclXL, overexpressed natural inhibitors of caspases like FLIP and XIAP, diminished MAP signaling pathway or deficiency in proteins involved in signal transferring from damaged DNA to cytoplasm, enhanced activity of repair mechanisms and efficient redox system are features mainly responsible for unsuccessful treatment with cisplatin [33]. Well defined molecular background of the resistance to cisplatin point out the way on how to surmount it. It was already known that some of combined treatments of cisplatin with other chemotherapeutics such as 5-fluorouracil improved therapeutic response rates in patients with head and neck cancer [71, 72]. Furthermore, inhibition of NER DNA repair system, cotreatment with histone deacetylase inhibitors (HDAC) such as trichostatin A (TSA) or suberoylanilide hydeoxamic (SAHA) [73], small molecules inhibitors of FLIP and XIAP as well as topoisomerase inhibitors strongly synergized with cisplatin, elevating its therapeutic potential.

## 2. Metallocenes in Anticancer Chemotherapy

Most of the metallodrugs used currently in chemotherapy treatment are based on platinum (cisplatin analogues), although as side effects are the weakest point in the use of cisplatin-based drugs in chemotherapy are the high number of side effects, many efforts are focused on the search of novel metal complexes with similar antineoplastic activity and less side effects as an alternative for platinum complexes. Transition-metal complexes have shown very useful properties in cancer treatment, and the most important work in chemotherapy with transition metals has been carried out with Group 4, 5, 6, 8, and 11 metal complexes.

From all the studied metal complexes, a wide variety of studies have been carried out for metallocenes which have become an alternative to platinum-based drugs.

According to the IUPAC classification metallocene contains a transition metal and two cyclopentadienyl ligands coordinated in a sandwich structure. These compounds have caused a great interest in chemistry due to their versatility which comes from their interesting physical properties, electronic structure, bonding, and their chemical and spectroscopical properties [74]. Academic and industrial research on metallocene chemistry has led to the utilization of these derivatives in many different applications such as olefin polymerization catalysis, asymmetric catalysis or organic syntheses, preparation of magnetic materials, use as nonlinear optics or molecular recognizers, flame retardants or in medicine [74].

Within medicine, metallocene complexes are being normally used as biosensors or as antitumor agents. Regarding their anticancer applicability, titanocene, vanadocene, molybdocene, and ferrocene have been traditionally used with very good results, however, recently also zirconocene derivatives have pointed towards a future potential applicability due to the increase of their cytotoxicity. All the other metallocene derivatives have been either not tested or have demonstrated no remarkable applicability in the fight against cancer. 

In this part of the paper, we will briefly discuss separately the properties of metallocene derivatives of titanium, zirconium, vanadium, molybdenum, and iron.

### 2.1. Titanocene Derivatives

**Figure 5:**
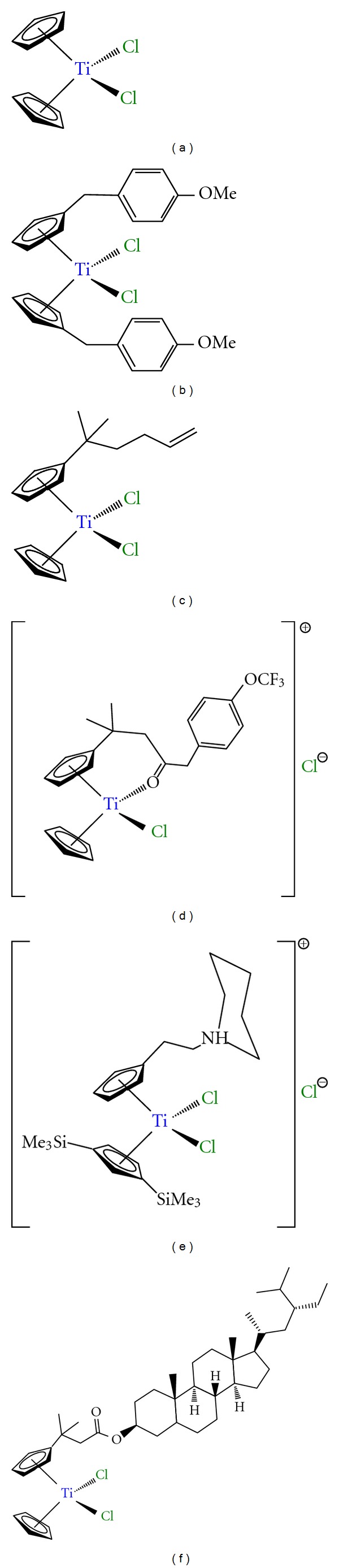
Titanocene derivatives used in preclinical and clinical trials: (a) titanocene dichloride; (b) titanocene-Y; (c) alkenyl-substituted titanocene derivative; (d) titanocenyl complex; (e) titanocene derivative with alkylammonium substituents; (f) steroid-functionalized titanocene derivative.

Titanocene derivatives are together with ferrocene complexes the most studied metallocenes in the fight against cancer. The pioneering work of Köpf and Köpf-Maier in the early 1980's showed the antiproliferative properties of titanocene dichloride, [TiCp_2_Cl_2_] (Cp = *η* ^5^-C_5_H_5_, Figure 5(a)). This compound was studied in phase I clinical trials in 1993 [75–77] using water soluble formulations developed by Medac GmbH (Germany) [78]. 

Phase I clinical trials pointed towards a dose-limiting side effect associated to nephrotoxicity which together with hypoglycemia, nausea, reversible metallic taste immediately after administration, and pain during infusion, seemed to be the weakest part of the administration of titanocene dichloride in humans. On the other hand, the absence of any effect on proliferative activity of the bone marrow, one of the most common dose-limiting side-effect of nonmetallic drugs, was in interesting result that increased the potential applicability of this compound in humans. 

Although phase I clinical trials were not as satisfactory as expected, some phase II clinical trials with patients with breast metastatic carcinoma [79] and advanced renal cell carcinoma [80] have been carried out observing a low activity which discouraged further studies.

However, after the recent work of many groups such as Tacke, Meléndez, McGowan, Baird, and Valentine the interest in this field has been renewed [81–85]. In this context a wide variety of titanocene derivatives with amino acids [86, 87], benzyl-substituted titanocene or *ansa*-titanocene derivatives [81], amide functionalized titanocenyls [88, 89], titanocene derivatives with alkylammonium substituents on the cyclopentadienyl rings [90–92], steroid-functionalized titanocenes [93], and alkenyl-substituted titanocene or *ansa*-titanocene derivatives (Figure 5) [94–96], have been reported with very interesting cytotoxic properties which enhance their applicability in humans. In particular, [Ti{*η* ^5^-C_5_H_4_(CH_2_C_6_H_4_OCH_3_)}_2_Cl_2_] (titanocene Y, Figure 5(b)) and its family, reported by Tacke and coworkers, have demonstrated to have extremely interesting anticancer properties which need to be highlighted.

**Figure 6:**
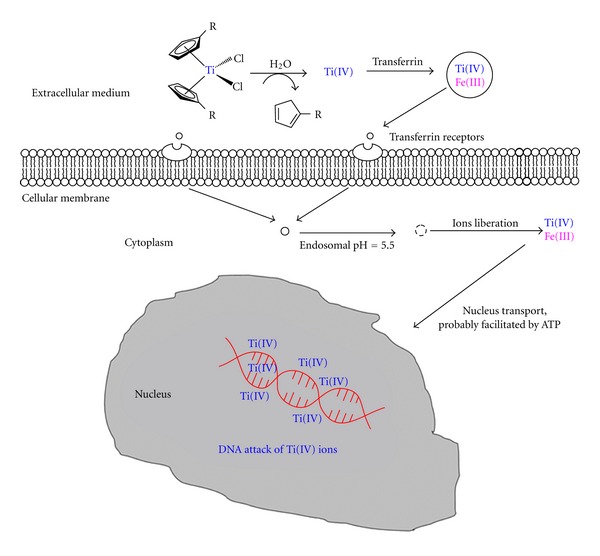
Proposed mechanism of action of titanocene derivatives (adapted from Abeysinghe and Harding, Dalton Trans. 32 (2007) 3474).

In general, the cytotoxic activity of titanocene complexes has been correlated to their structure, however, there are still several questions regarding the anticancer mechanism of titanocene(IV) complexes. According to the reported studies in the topic, it seems clear titanium ions reach cells assisted by the major iron transport protein “transferrin” [97–100], and the nucleus in an active transport facilitated probably by ATP. In a final step, binding of titanium ion to DNA leads to cell death (Figure 6) [101, 102]. However, recent experiments have shown interactions of a ligand-bound Ti(IV) complex to other proteins or enzymes [103–105], indicating alternatives in cell death mechanisms, which is currently leading to intensive studies by several research groups.

### 2.2. Zirconocene Derivatives

**Figure 7:**
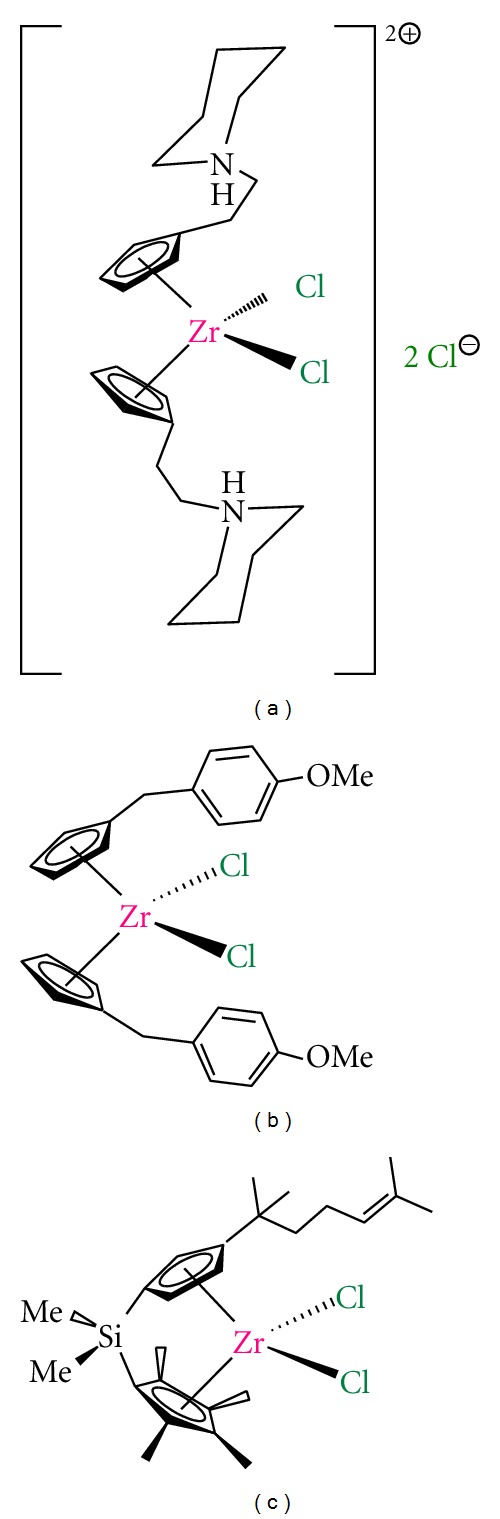
Zirconocene derivatives with anticancer activity: (a) zirconocene derivative with alkylammonium substituents; (b) zirconocene-Y; (c) alkenyl-substituted *ansa*-zirconocene complex.

An alternative to titanium complexes may be zirconium(IV) derivatives which are in a very early stage of preclinical experiments. Already in the 1980's Köpf and Köpf-Maier showed the potential of zirconocene derivatives as anticancer agents and very recently, two different studies on zirconocene anticancer chemistry have been reported [106, 107]. These studies by Allen et al. [106] and Wallis et al. [107]have described the cytotoxic activity of different functionalized zirconocene complexes, observing an irregular behavior in the anticancer tests, from which only the complexes [Zr{*η* ^5^-C_5_H_4_(CH_2_)_2_N(CH_2_)_5_}_2_Cl_2_ *·*2HCl] (Figure 7(a)) and [Zr{*η* ^5^-C_5_H_4_(CH_2_C_6_H_4_OCH_3_)}_2_Cl_2_] (zirconocene Y, Figure 7(b)) have shown promising activity that needs to be improved in order to apply them in anticancer chemotherapy.

In parallel, our research group reported the synthesis, structural characterization, catalytic behavior in the polymerization of ethylene and copolymerization of ethylene and 1-octene and the cytotoxic activity on different human cancer cell lines of a novel alkenyl substituted silicon-bridged *ansa*-zirconocene complex (Figure 7(c)) which proved to be the most active zirconocene complex on human A2780 ovarian cancer cells, reported to date [108].

There is still hard work to do in this field to find a suitable zirconocene complex with increased cytotoxic activity and good applicability in humans.

### 2.3. Vanadocene Derivatives

Vanadocene dichloride, [VCp_2_Cl_2_] (Cp = *η* ^5^-C_5_H_5_), was extensively studied in preclinical testing against both animal and human cancer cell lines, observing a higher *in vitro* activity of vanadocene(IV) dichloride on direct comparison with titanocene(IV) dichloride [109–111].

**Figure 8:**
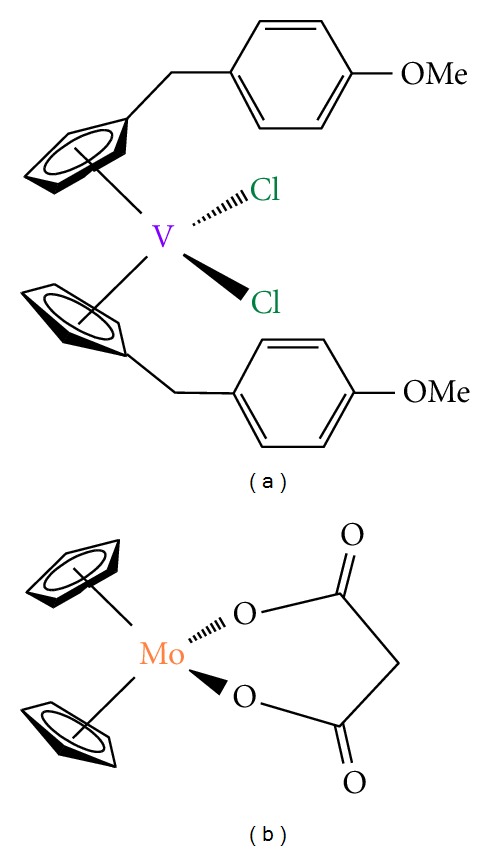
(a) Vanadocene-Y; (b) molybdocene carboxylate derivative.

These results encouraged further preclinical studies which were restarted around eight years ago [112–114], and have been recently extended [115–118] with the study of the cytotoxic properties of vanadocene Y (Figure 8(a)) and similar derivatives. In addition, a comprehensive study of the cytotoxic activity of methyl- and methoxy-substituted vanadocene(IV) dichloride toward T-lymphocytic leukemia cells MOLT-4 has also been recently reported [119]. In most cases, vanadocene derivatives are more active than their corresponding titanocene analogues, however, the paramagnetic nature of the vanadium center, which precludes the use of classical NMR tools, makes the characterization of these compounds and their biologically active species more difficult. The need of the use of X-ray crystallography and other methods such as electron-spin resonance (ESR) spectroscopy slows down their analysis and the advances in this topic.

### 2.4. Molybdocene Derivatives

After the work of Köpf and Köpf-Maier there were some evidences of the potential properties as anticancer agents of molybdocene dichloride derivatives. In recent years, the extensive work carried out by many different research groups confirmed the anticancer properties of molybdocene [120–124]. But not only the cytotoxic properties of these compounds have been reported, the hydrolysis chemistry of [MoCp_2_Cl_2_] has been intensively studied [125–127]. In the case of molybdocene derivatives the stability of the Cp ligands at physiological pH has led to the study of many different biological experiments with results which show new insights on the mechanism of antitumor action of [MoCp_2_Cl_2_] and some analogous carboxylate derivatives (Figure 8(b)) [117, 128–130]. 

### 2.5. Ferrocene Derivatives

The discovery of the cytotoxic properties of ferricinium salts on Ehrlich ascite tumors by Köpf and Köpf-Maier [131, 132] were an early breakthrough for the subsequent development of novel preparations of this class of anticancer agents. 

There are different groups working in this field, however, to date, the most interesting work in the field of anticancer applications of ferrocene derivatives is being carried out by Jaouen and coworkers. 

**Figure 9:**
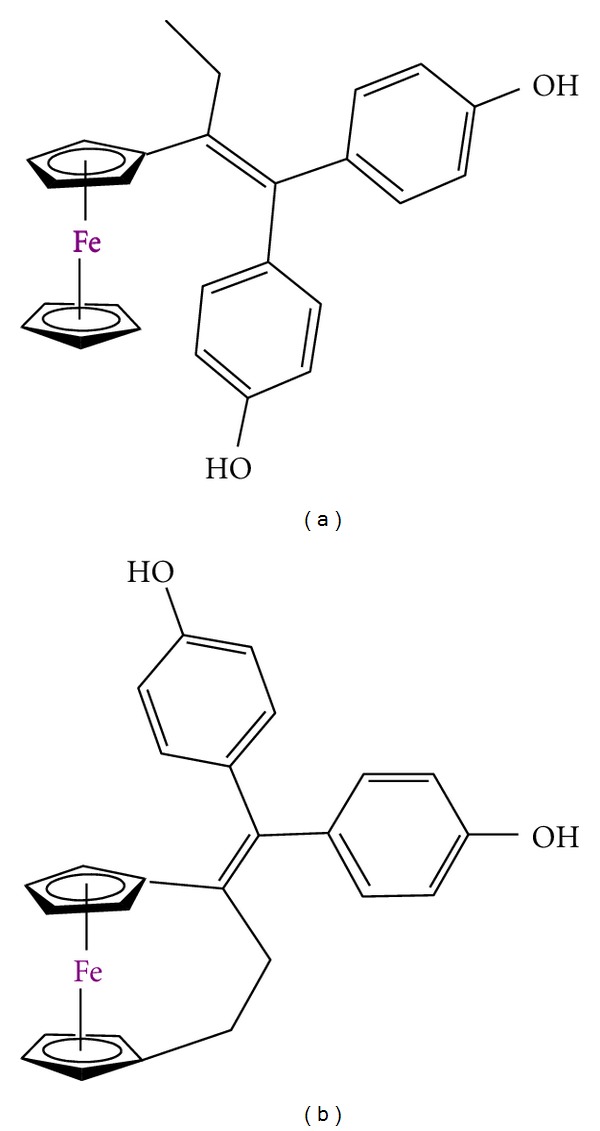
Ferrocene derivatives used in preclinical trials: (a) hydroxyferrocifens; (b) ferrocene complex with a [3] ferrocenophane moiety.

This group has published several reports on the synthesis of novel functionalized ferrocene derivatives “hydoxyferrocifens” which consist of the linking of the active metabolite of tamoxifen and ferrocene moieties (Figure 9(a)) [133, 134]. This novel class of compounds are able to combine the antioestrogenic properties of tamoxifen with the cytotoxic effects of ferrocene [135–137]. From all these complexes, the outstanding cytotoxicity of a ferrocene complex with a [3] ferrocenophane moiety conjugated to the phenol group (Figure 9(b)) is important to be remarked [138].

In addition, ferrocene-functionalized complexes with steroids or nonsteroidal antiandrogens have also been reported to be very effective to target prostate cancer cells [139].

But not only the design and synthesis of novel ferrocene derivatives with different ligands and cytotoxic properties have been studied, several investigations on the cell death induced mechanism of these anticancer drugs have been reported. Thus, two different action mechanisms have been proposed for ferrocene derivatives, production of electrophilic species, and/or production of ROS species [140].

### 2.6. Future Tendencies in the Use of Metallocenes in Anticancer Chemotherapy

Almost all metallocene derivatives which have been studied either in preclinical or clinical trials are extremely hydrophobic to be intravenously administered, thus limiting their bioavailability for clinical applications. 

Novel formulations of metallocene derivatives in macromolecular systems such as cucurbit(n)urils [140] or cyclodextrins [141] leading to a presumably higher applicability in humans.

In addition, using a different approach, but with the same goal of circumventing the solubility problems of metallocenes in biological media, several metallocene-functionalized MCM-41 or SBA-15 starting from different titanocene dichloride derivatives with anticancer activity have been reported and may be a good starting point for the development of novel metallocene-based drugs for the treatment of bone tumors [142–145]. 

## 3. Conclusions

One of the most potent antitumoral drugs cisplatin deserves special attention as exceptional of few with healing effect. Important role in the action of cisplatin is interaction with nuclear DNA and unfeasibility of the cell response to repair DNA strain containing covalently bonded dichloridoplatinum(II) moiety (nucleotide excision repair mechanism). Beside DNA, cisplatin might interact with other biomolecules (thioproteins, RNA) and in that way could be deactivated or even may possibly tune different signaling pathways involved in mediation of cell death, which is cell type specific. Namely, cisplatin has intense effects on signaling pathways facilitated by MAPs (e.g., ERK, JNK, p38). In recent years information on the cellular processing of cisplatin has essentially arisen. Knowledge collected from studies about biological effects of cisplatin and development of cisplatin resistant phenotype afford important clues for the design of more efficient and less toxic platinum and nonplatinum metal based drugs in cancer therapy. It is to be expected that nonplatinum metal compounds may demonstrate anticancer activity and toxic side effects noticeably different from that of platinum based drugs. Thus titanocene, vanadocene, molybdocene, ferrocene, and zirconocene revealed encouraging results in *in vitro* studies. These compounds might enter by different transport mechanism through cell membrane and distinctly interact with biomolecules than cisplatin. Notwithstanding the extensive applications of cisplatin in the new investigations will provide us with powerful facts for finding a novel efficient and nontoxic metallotherapeutics in anticancer treatment.
